# What factors are associated with dental general anaesthetics for Australian children and what are the policy implications? A qualitative study

**DOI:** 10.1186/s12903-018-0638-8

**Published:** 2018-10-24

**Authors:** John Rogers, Clare Delany, Clive Wright, Kaye Roberts-Thomson, Mike Morgan

**Affiliations:** 1 University of Melbourne, Melbourne, Victoria Australia; 2 University of Sydney, Sydney, New South Wales Australia; 3 University of Adelaide, Adelaide, South Australia Australia

**Keywords:** Dental caries, Children, Dental general anaesthetics, Potentially preventable dental hospitalisations

## Abstract

**Background:**

Dental general anaesthetics undertaken on young children are amongst the most common of all potentially preventable hospitalisations of children in Australia. They are costly for families and the community and entail some risk. The aim of the study was to explore the views of stakeholders about factors associated with children’s dental general anaesthetics in Victoria, Australia and to identify policy implications.

**Methods:**

Interviews with stakeholders were used to develop a framework of factors. Interview data were subject to qualitative analysis, informed by Interpretative Phenomenological Analysis.

**Results:**

Eight themes that encompassed 30 main factors were identified through focused discussions with 16 stakeholders. While the safety of dental general anaesthetics has improved and mortality rates are low, side effects are common. Push factors for children’s dental general anaesthetics include a perceived greater ‘child-focus’; preferred models of care; low oral health literacy; parent guilt; convenience; and some dentists reluctance to treat high needs children in the clinic. Factors that may decrease the prevalence of dental general anaesthetics include: prevention of dental caries; using alternative approaches; an appropriate workforce mix; enhancing oral health literacy; and development of guidelines.

**Conclusion:**

The prevalence of hospitalisation of children to treat dental caries is increasing. Many factors influence the prevalence of paediatric dental general anaesthetics - relating to the child, parent, oral health professional, financial impact, health risk, and accessibility to facilities. There are quality of care and convenience benefits but also high costs and possible health risks. Family, workforce and health system factors have been identified that could decrease the prevalence of paediatric dental general anaesthetics.

## Background

Dental general anaesthetics (DGAs) undertaken in hospitals or day procedure centres on young children are amongst the most common of all hospitalisations of children in Australia. Of the 130,792 DGAs in 2013–14, 20,607 were 0–9 year-olds hospitalised for potentially preventable reasons [1]. The number of Potentially Preventable Dental Hospitalisations (PPDHs) in this age group has been increasing. Between 2013-14 and 2015–16 there was a 17% increase to 4891 in Victoria, making PPDHs the highest of all Potentially Preventable Hospitalisations (PPHs) and double the rate for asthma admissions [2]. These PPHs are admissions for conditions where hospitalisation is considered to have been avoidable if timely and adequate non-hospital care had been provided [1] or if the condition had been prevented in the first place. In 2015–16 the principal diagnosis for over 90% of PPDHs in children was dental caries [3], meaning that almost all DGAs were PPDHs. Just over half of children’s PPDHs (53%) were conducted in public hospitals in Victoria in 2015–16 [4].

Reducing PPHs is an objective in health care reform with the aim of improving patients’ outcomes, reducing pressure on hospitals and enhancing health system efficiency and cost effectiveness [5]. PPDHs are one of 11 PPHs that are included in the National Healthcare Agreement [6] between the Australian Government and the state and territory governments as a performance indicator [7]. Reducing the rates of PPDHs is a key strategy in the Australian National Oral Health Plan 2015–2024 [8].

The most recently published guidelines for indications for DGA for children and adolescents have been developed by the American Academy of Pediatric Dentistry [9]. Indications are proposed for: patients who cannot cooperate due to lack of psychological or emotional maturity and/or mental, physical or medical disability; patients for whom local anaesthesia is ineffective because of acute infection, anatomic variations, or allergy; the extremely uncooperative, fearful, anxious or uncommunicative child or adolescent; those requiring significant surgical procedures; patients for whom the use of general anaesthesia may protect the developing psyche and/or reduce medical risk; and patients requiring immediate, comprehensive oral/dental care [9]. Higher quality dental care can often be provided in a DGA because it is easier to manage saliva and the tongue in the more controlled environment. A systematic review of 20 studies from 14 countries concluded that dental care under a GA improves the overall quality of life of children with high levels of parental satisfaction [10].

Concerns about DGAs for children include the family and health system costs and mortality and morbidity risks. The average cost of a DGA for under 15 year-olds in Western Australia has been estimated at $2039 and $5234 when indirect costs are added [11]. Richardson and Richardson estimated that the cost of the 50,000 PPDHs in Australia in 2008–09 was $233 million, an average cost of $4660 [12]. There is little information published on the morbidity and mortality impacts of DGAs in Australia. The overall GA mortality rate in children was estimated to be 1:150,000 in 2005 [13]. Knapp and colleagues systematic review quoted above found that some quality of life sub scales worsened after a DGA [10]. Qualitative research has also found that some children report that they were scared during the DGA process [14] while parents have reported being shocked at the amount of blood and the behavioural state of their child [15].

There may be variations in views among Oral Health Professionals (OHPs) about indications for conducting DGAs in children. DGA guidelines from the United Kingdom indicate that the majority of dental services for children can be carried out using either local anaesthesia or local anaesthesia with conscious sedation, and that patient/carer preferences are a condition that rarely justifies a GA [16]. United Kingdom guidelines for people living with learning disabilities state that a DGA for people with a disability should be the ‘*last choice of treatment’* [17]. In the Australia context, Alcaino and colleagues have noted that *‘although most children will cope with dentistry in the normal setting, many more may benefit from delivery of extensive dentistry in one session under GA’* [13]. In the Australian public dental system there have been indications that children who are considered difficult to treat in the dental chair are referred for a DGA without consideration of alternative care [18]. The most recent Australian DGA guidelines for children were developed by the Australasian Academy of Paediatric Dentistry in 2002 and published in 2005 [19]. There are no standard Australian policy guidelines for referral for publicly funded DGAs.

The literature that has examined factors which influence dental hospitalisation of children is generally descriptive and is predominantly derived from cross sectional studies which limit the ability to ascribe causality. Few studies have measured the strengths of associations or controlled for potentially confounding factors. Information is available on DGA frequency [1, 20], individual characteristics of those who have a DGA [11, 21, 22], and the impact of environmental factors such as access to water fluoridation and primary dental care [23]. However, little is known about the factors that drive the decisions of OHPs when they encounter a child in their clinic including how they make decisions about the most appropriate treatment for a particular dental problem. There are also few references in the literature to DGA mortality in Australia or the access to operating theatres.

The decision to perform a DGA is potentially influenced by the perspectives and clinical reasoning of the individual oral health professional and more broadly by drivers within the health and education systems. Identifying these factors, including how they intersect, is necessary for the development of strategies to reduce the rates of PPDHs as called for in the Australian National Oral Health Plan 2015–2024 [8].

Therefore the aim of this study was to explore the views of stakeholders in the public dental health system about factors associated with children’s DGAs and to identify policy implications. The main research question was ‘What is the perception of OHPs and Hospital Admission Decision Makers (HADMs) about factors associated with the frequency of DGAs among children in Victoria, Australia?’

## Methods

The research design was qualitative drawing from interpretivism [24] and phenomenology [25]. These methodologies are appropriate when the research aims to understand people’s interpretations, perceptions, perspectives and experiences of a particular situation (or phenomenon). These research paradigms acknowledge that people experience and understand the same ‘objective reality’ in very different ways, which then shapes their decision making [26].

A two stage process was used to collect data. The first stage involved interviewing OHPs and HDAMs working in the public sector to identify factors which might influence decisions to perform a DGA for a child. These data were analysed and a framework of factors influencing DGAs developed.

The framework was then used in stage two of data collection. Paediatric dentists were interviewed using the framework to encourage these participants to reflect on their own decisions and the influences they had encountered. Paediatric dentists undertake the majority of DGAs in Victoria. Participants either agreed or disagreed with the identified factors and added their perspective. In-depth interviews were also held with HDAMs to explore factors relating to health system policy and practice of dental hospitalisation in Victoria.

Stage one discussions took place between the chief investigator (JR) and 11 OHPs and two HDAMS. Discussions were held between August 2012 and May 2014 and lasted from 30 to 90 min. Notes were taken and discussed with the participants. Memos or ‘notes to self’ on emerging categories and ideas were written during the discussions. Thematic analysis was undertaken [26] and the framework developed. Participants were purposefully selected [27] on the basis of their experience and expertise related to DGAs in Victoria particularly in the public sector. The OHPs included four paediatric dentists, two dentists, two dental therapists, and three dental public health specialists as listed in Table 1. Both HDAMs were senior public hospital executives. All of the stakeholders who were approached agreed to participate.

**Table 1:** Study participants

	Number	Years of experience related to DGAs (average)
Preliminary discussions		
Oral health professionals (OHPs) – 4 paediatric dentists, 2 dentists, 2 dental therapists and 3 dental public health specialists.	11	28 years
Hospital admission decision maker (HADMs)	2	
In-depth interviews		
Paediatric dentists	3	23 years
HADMs	3	

In the stage two data collection, in-depth interviews were conducted with three of the paediatric dentists and three HADMs. The HADMs included two Department of Health policy makers and a senior public hospital executive as shown in Table 1. All participants were purposefully selected on the basis of positions they hold in the public sector provision of dental general anaesthesia in Victoria. The paediatric dentists also had private practice experience. The framework and research questions are presented in the Results.

The in-depth interviews were conducted by the chief investigator (JR) from May 2014 to June 2015. Participants were provided with a plain language statement about the research and signed consent forms. Open-ended questions were used to allow the participants to add their interpretation and understanding of what they thought was important. The semi-structured nature of the discussions encouraged participants to respond more freely than a structured interview format [24]. Interviews generally ran from 60 to 90 min. Comprehensive notes were taken during the interviews. These notes were compiled and provided to the participants to check their accuracy.

### Analytical plan for data

Interview data from both stages were subject to qualitative analysis, informed by Interpretative Phenomenological Analysis (IPA) [28]. Analysis of data within the IPA approach is flexible but with general procedures that define the approach and ensure its rigor. Procedures followed in stage one were: JR reading and re-reading the notes of discussions with OHPs and HDAMs to identify the matters or themes that were raised by participants; annotating notes with the themes that arose; reviewing the memos written during the interviewing process to support an understanding of the concepts within the data; and clustering participant themes. Notes and themes were then reviewed by MM and CW and consensus reached on main and subordinate themes.

A framework was developed using the identified themes of factors associated with DGAs in children. The framework was then used in stage two. Notes of the interviews were compiled as for the discussions with the OHPs and HDAMs and as mentioned, provided to the participants to check their accuracy. Verbatim quotes were checked in this way. Themes were then written up using selected participant statements with interpretative commentary. These steps were followed by preparing a discussion of identified themes against what is presently known in the literature and reflection on the research to identify policy implications and further areas for research.

## Results

Discussions were held with 11 OHPs and two HDAMs to develop the framework. While the sample was only a relatively small proportion of the dental and hospital policy professionals in Victoria, the most influential people associated with dental hospitalisation in the public sector were included. The average years of relevant experience was 28 years, as shown in Table 1.

A thematic analysis of the discussions identified eight key themes that encompassed 30 main factors that the participants considered are relevant to understanding the frequency of dental hospitalisation of children in Victoria (Table 2). Themes (T) were: T1, criteria for DGAs; T2, child factors; T3, dental provider factors; T4, parent factors; T5, risk; T6, financial impact; T7, access to general anaesthetic facilities; and T8, treatment provided. Theme 3, dental provider factors, comprised the sub themes of paediatric dentist factors, general dentist factors, and dental therapist factors.

**Table 2:** Framework of themes and factors associated with Dental General Anaesthetics (DGAs) in children in Victoria

Themes	Main associated factors
1. Criteria for DGAs	• Importance of preventing dental caries • Variation in views about appropriate threshold for paediatric DGAs • Perceived increase in dental caries prevalence and dental care needs • Change in attitude by OHPs to DGAs • Limitation of existing guidelines
2. Child factors	• Greater emphasis on children’s attitude toward dental treatment • Possible change in behavioural management techniques
3. Dental provider factors	
3.1. Paediatric dentist factors	• Quality of care • Case selection for DGAs
3.2. General dentist factors	• Reluctance to treat young children • Changes in general dental practice • Limited alternatives to DGAs
3.3. Dental therapist factors	• Increased employment in private practice • Role as primary oral health care workers
4. Parent factors	• Convenience of DGAs • Parenting styles • Oral health literacy • Guilt • Cultural variations
5. Risk	• Improved safety of DGAs • Mortality • Morbidity
6. Financial impact	• Private sector DGA cost • Public sector DGA cost
7. Access to general anaesthetic facilities	• Economic efficiencies • Dental provider issues • Department of Health policy factors • Rural hospital issues.
8. Treatment provided and follow-up after a DGA	• Emergency and elective DGAs • Appropriate dental treatment under DGAs • Follow up prevention appointments

Questions for the in-depth semi structured interviews in stage two of the data collection were based on the framework and are presented in Table 3. The questions were different for the paediatric dentists and HADMs to reflect these groups different roles in relation to DGAs. The in-depth interviews confirmed that the themes in the framework were relevant. Further detail emerged from these interviews.

**Table 3:** Interview questions

Questions for paediatric dentists ‘What do you consider to be the key factors related to DGAs in Victorian children?’ Prompts: • Criteria for DGAs • OHP factors • Parent and child factors • Indications for DGAs • Access to DGA facilities • DGA costs • DGA risks.	
Questions for Hospital admission decision makers (HADMs) • ‘What is the current policy, associated guidelines and practice related to access to hospital theatres for DGA?’ • ‘Have the current policy/guidelines/practice changed?’ Prompts: • Funding changes • Any difference in factors for metropolitan and rural hospitals. • Influence of waiting lists.	

### Theme 1 criteria for DGAs

All participants noted that it was preferable to prevent dental caries rather than have to treat it. While all participants indicated that a DGA was required for higher quality outcomes for young children with extensive or complex dental treatment needs and/or behavioural issues, there were variations in views about the appropriate threshold. A commonly mentioned indicator from paediatric dentists was if the child required treatment in all four quadrants of the mouth. It was noted that some operators have a lower DGA threshold such as when a child will not tolerate the placement of rubber dam. Several OHPs spoke of peers in the public and private sector who tended to use DGA as a ‘first resort’ or as the ‘default choice’.

Four of the five paediatric dentists believed that there had been an increase in dental caries prevalence in Victorian children since 2000 with a consequent increase in dental treatment needs. None of these participants had readily available data to quantify this perceived change.

Four paediatric dentists spoke of the change in attitudes toward DGAs by general and paediatric dentists.

*‘Twenty years ago, treating a young child under GA was generally seen as a failure by clinicians to manage care in the dental clinic. Fewer hold this view now’.* (Paediatric dentist)

No published Australian guidelines on criteria for DGAs were acknowledged as best practice by any of the participants.

*‘The Australasian Academy of Paediatric Dentistry published guidelines in 2005 but there have not been any updates’.* (Paediatric dentist)

Participants working in the public system noted that there were no standard Australian policy guidelines for referral for publicly-funded DGAs.

*‘It would be fantastic to establish a national group to agree on guidelines that can be accepted at a national level’.* (Paediatric dentist)

### Theme 2 child factors

There was agreement that there is a greater emphasis on the child’s attitude toward dental treatment compared to 30 years ago. The child is now more likely to be asked than told what they want or need. Behavioural management techniques used in dentistry appear to have changed.

*‘There is now less acceptance by children, parents and dental providers of ‘chasing the child’ and ‘rough and tumble’ in the dental chair’.* (Paediatric dentist)

A paediatric dentist with experience working in a children’s hospital noted that there has been a general increase in ‘child focus’ across all health disciplines.

*‘The accepted best practice is ‘do not stress the child’.* (Paediatric dentist)

A ‘pain free’ policy has led to a shift to use general anaesthesia in the hospital for procedures previously undertaken under local anaesthesia. For example, short general anaesthesia is now used in medical procedures such as lumbar punctures in order to be kinder (‘less stressful’) to the child. Nitrous oxide (relative analgesia) sedation is also now in widespread use in hospital medical clinics and its use has also increased in the hospital dental clinics.

### Theme 3 dental provider factors

#### Paediatric dentist factors

The two main areas relating to paediatric dentists identified by participants were quality of care and case selection for DGAs. Case selection responses have been included under Theme 1 Criteria for DGAs.

Four of the five paediatric dentists indicated that a DGA can be the preferred model of care.

*‘Higher quality treatment is possible, especially for preschool children’*. (Paediatric dentist)

*‘It can be more predictable with less complications’.* (Paediatric dentist)

*‘A DGA for a child can be a preferred model of care for a quality outcome and ability to manage all treatment’.* (Paediatric dentist)

These informants considered that treatment under a DGA can be less haphazard and provide the most efficient outcome. Dental treatment completed in a one-hour DGA session could require four to six appointments in a dental clinic. It was noted that the quality of care possible also depends on the length of the GA session.

Two of the paediatric dentists stated that undertaking a DGA does not necessarily allow higher quality care to be provided, nor does it improve the chances of being able to provide subsequent care to children in a dental clinic. It was noted that under a GA it is difficult to obtain an x-ray and it may not be possible to check the occlusion. In addition, quality can be compromised because of mouth rather than nasal intubation. It was reported that many anaesthetists do not like using nasal intubation. A paediatric dentist referred to research about DGAs and their impact on a child’s attitude to future dental care.

*‘DGAs don’t necessarily reduce the anxiety a child may have about receiving dental treatment in a dental clinic in the future’.* (Paediatric dentist).

#### General dentist factors

All of the clinician participants (the paediatric dentists, general dentists, and dental therapists) considered that general dentists may be reluctant to treat young children due to a lack of confidence because they may not have had the necessary training or experience. A general view was that there was not sufficient practical experience during basic dental degree training. For example, a paediatric dentist noted that dental students may not have the opportunity to give a block local anaesthetic to a child, or learn about alternative sedation techniques to DGAs such as using relative analgesia.

It was reported that there can be a shortage of appropriate patients for dental students to be able to develop their behavioural management and clinical skills. Many children who attend student clinics do not require care beyond examinations and the placement of fissure sealants. It was also noted that training in relative analgesia and also intravenous sedation is limited in Victoria.

*‘It is a tragic loss of opportunity that there is no course of training in intravenous sedation available in Victoria’. ‘There is a desperate need for a wider range of options than avoidance of treatment or full general anaesthesia’.* (Dentist)

Three participants raised an issue about perception of the professional status of dentists.

*‘If a general dentist is having difficulty managing the dental care of a child, he or she may refer the child to a paediatric dentist in order to save face with parents’.* (Dentist)

Participants mentioned changes in general dental practice that have affected DGA rates such as more referral options with increased numbers of registered paediatric dentists and less acceptance by children, parents and dental providers of ‘rough and tumble’ in the dental chair.

#### Dental therapist factors

The dental therapist participants, in common with other OHPs as mentioned under T1, indicated that it was preferable to prevent dental caries rather than treat it and that DGAs were required in certain circumstances. Several participants noted that more private practices are employing dental therapists who manage most of the dental care for children. Participants involved in education or service delivery noted that oral health and dental therapist training focuses on managing the general clinical care of children and that they were efficient and effective primary oral health care workers.

The dental therapist participants spoke of cases where they were asked to provide a second opinion after parents had been told by another OHP that their child would require a DGA. In these cases the dental therapists reported that they were able to manage the child in the dental chair. Therapists acknowledged that paediatric dentists had advanced skills and would refer children to them if the treatment required was outside a therapists scope of practice.

### Theme 4 parent factors

While parents were not interviewed, most study participants commented on parent factors that affected the frequency of DGAs. Comments were made about convenience, parenting styles, oral health literacy, guilt, and cultural variations.

Participants noted that some parents consider a DGA to be convenient because treatment can be completed in one session, rather than three or more visits to a dental clinic. This reduces the time needed for parents to take time off work in addition to potentially there being less expense for childcare for other children.

*‘A DGA is attractive to parents who are time-poor and asset-rich’.* (Paediatric dentist)

Some respondents framed parents’ requests as an example of ‘permissive’ parenting styles compared to ‘authoritative’ or ‘authoritarian’ styles, which may have led to an increase in DGAs as parents were less likely to insist on their child ‘behaving in the dental chair’.

Comments were made about the wide variation in oral health literacy among parents. Several participants mentioned that parents accessing private care for their child generally had higher oral health literacy, but some were influenced by anti-fluoride articles they had accessed on the internet. These parents were more likely to use herbal toothpastes that do not have the dental caries prevention impact of fluoride toothpastes. Three paediatric dentists also mentioned the variation in the perceived importance of primary teeth to parents.

*‘Some parents do not value primary teeth’.* (Paediatric dentist).

Some parents were not aware of the impact of diet on oral health. Others were, but considered it too difficult to change feeding habits and a DGA was the price they were prepared to pay.

Several paediatric and general dentists noted that parents can become defensive when issues of diet are raised, possibly because of feelings of guilt that their child had dental caries.

*‘Some parents feel guilty that their child has dental caries’.* (Paediatric dentist)

Several paediatric dentists noted that more parents are asking that white fillings rather than silver crowns are used to cap decayed molar teeth because these parents are concerned that visible dental work was a sign of parental neglect. A general dentist commented that there may be occasions when an OHP could ‘trade on’ parental guilt when discussing the need for a DGA.

Several OHPs noted that some parents are reluctant for their children to have care under a DGA. Cultural variations in parental attitudes toward DGAs were raised.

*‘There are cultural variations, for example some parents with Asian backgrounds are more concerned about the risks of a DGA’.* (Paediatric dentist).

### Theme 5 risk

When paediatric dentists mentioned risk, they indicated that the safety of a DGA had improved with new anaesthetic drugs and better monitoring. The newer anaesthetic agents have fewer side effects. With better monitoring, problems are detected earlier when intervention can occur. Nausea was noted by some paediatric dentists as a still common side effect.

Paediatric dentists noted that DGA-linked child deaths were rare in Australia. The fatalities mentioned were limited to the death of a young child in Broken Hill in 1998 and a fatality in a Brisbane dental clinic more than 20 years ago. Both deaths were associated with the children’s underlying medical problems.

### Theme 6 financial impact

Participants mentioned costs to the family and to the health system. Paediatric dentists reported that costs ranged from $2500 to $8000 in the private sector, with most admissions costing between $3500 and $5000.

*‘In the private sector there are three direct cost components to a DGA: the anaesthetist’s fee, clinician’s fee and facility bed fee’.* (Dentist).

Participants noted that costs are predominantly ‘out of pocket’, with Medicare reimbursement only available for part of the anaesthetist fee.

*‘Private insurance rebates may cover only about half of the DGA costs’.* (Dentist).

Hospital administrators advised that the national Independent Hospital Pricing Authority determined through a survey of public hospitals that the average cost for the most common DGA (dental extractions and restorations) was $3029 in public hospitals in 2012–13 [29]. These direct costs do not include indirect costs such as childcare for other children and loss of income from taking time off work.

### Theme 7 access to DGA facilities

Four issues concerning access to operating theatres were identified from participants’ responses: economic efficiencies, dental provider issues, Department of Health and Human Services policy, and rural hospital issues.

Several participants who had worked in the private hospital system noted that general dental treatment of children was perceived as less economically viable for a facility than extraction of wisdom teeth, the placement of grommets, or the removal of tonsils and adenoids.

*‘Whereas a dental clinician may see three to five children in a three-hour session, a maxillofacial surgeon could extract the wisdom teeth of six patients, grommets could be placed in the ears of six children, or 10–15 cataract operations could be performed’.* (Paediatric dentist).

Participants with experience in the public system commented that a DGA can be a lower priority than a general anaesthetic for procedures that are monitored for waiting times by the Department of Health and Human Services and publically reported. Using the limited theatre resources for DGAs means that less time is available for procedures for which waiting times are reported publicly. Respondents commented that in busy hospitals there was a pressure on access to operating theatres.

*‘There is never enough theatre time’*. *‘If it is not counted (for waiting list reporting) it does not count’.* (Hospital admission decision maker)

Paediatric dentists noted that access to operating theatres was more difficult in the public system compared to the private system. However, they did note that some paediatric dentists and general dentists complained about poor access to private facilities for DGAs. Both an experienced general dentist and a hospital manager said that clinicians who appreciated that private hospitals and day procedure centres need to make a profit, and arranged their cases and length of treatment accordingly, appeared to have little problem accessing theatres.

The impact on DGA rates of hospital admission policy changes was raised by HDAMs. An example was a change in policy by the then Victorian Department of Health in 2011. Patients who attended emergency departments could not be recorded as an admission if they were admitted directly to a public hospital bed. A HDAM noted that total public hospital admissions decreased in 2012–13 compared to 2011–12, but then increased again in 2013–14 as hospitals established different arrangements for this group.

Two HDAMs indicated that there may be fewer barriers to DGAs in smaller rural hospitals, compared to larger metropolitan facilities. This is because small rural hospitals in Victoria are globally funded, not casemix funded, and have fewer medical specialty services competing for theatre time. It was noted that rural hospitals can have a regular general anaesthetic session for local dentists, that is, a ‘dental list’ every week or month. Where this occurs, there is some pressure on dentists to use these sessions and not risk losing them which may reduce access for providing timely DGA care for high needs cases.

### Theme 8 treatment provided and follow-up after a DGA

Paediatric dentists explained that emergency DGA cases, where a child has an infected tooth causing a swollen face, can take less than 15 min in theatre because only the teeth that are causing pain or infection and grossly decayed teeth are extracted. Non-emergency sessions are generally longer and more comprehensive treatment is provided.

The need to provide appropriate dental treatment under DGA was mentioned by most paediatric dentists. Paediatric dentists spoke of the need for ‘aggressive treatment’ to reduce the prevalence of repeat DGAs. Most interviewees indicated the need to discuss oral hygiene and diet with the family after their child’s DGA to reduce the likelihood of repeat DGAs for the child or their siblings.

## Discussion

This research has explored the views of OHPs and HADMs about factors associated with the frequency of DGAs among children. The in-depth interviews with paediatric dentists and HDAMs confirmed that the themes in the framework developed from initial focused discussions with participants were relevant. A wide range of factors were identified. These can be organised into ‘push’ factors for DGAs and potential factors to decrease DGA rates.

### Push factors for children’s DGAs

It is evident that approaches to the dental hospitalisation of children have been changing as a result of societal, technical and dental provider influences. Seven factors were identified that may increase DGA prevalence: a perceived greater ‘child-focus’ with emphasis on not stressing the child; a perceived increase in dental caries in children; DGAs as a preferred model of care; parents low health literacy; parent guilt; convenience for parents; and some dentists reluctance to treat children because they have not had the training and/or experience. These factors will be discussed before addressing the identified factors that are likely to decrease DGA prevalence.

The impact of a perceived greater ‘child-focus’ on the frequency of DGAs is difficult to quantify. Several participants noted that there had become a greater emphasis in ‘not stressing the child’ which was possibly associated with more permissive rather than ‘authoritative’ or ‘authoritarian’ parenting styles. There was a general view amongst OHPs that there is now less acceptance by parents, children and OHPs of ‘rough and tumble’ in the dental chair. It was noted by a paediatric dentist that this approach was in keeping with a more pain free policy that has been adopted in general health care.

Four of the five paediatric dentists stated that they considered that children’s dental caries rates and consequently dental treatment needs had increased in Victoria since 2000. High dental treatment needs have been shown to be a key driver of DGAs [30]. There is a lack of population data on trends in children’s oral health in Australia to test this perception. The results of the only two child population oral health surveys that have been conducted in Australia indicate that dental caries rates in 5–6 year olds have remained similar between 1987 and 88 and 2014–16, with significant reductions in rates in 12 year-olds [31]. However the children accessing paediatric dentists would more likely be high risk children and not representative of the general population.

Jamieson and Roberts-Thomson have suggested that one possible reason for the increased rate of dental hospitalisation of Australian children between 1997 and 98 and 2003–2004 was the increased number of paediatric dentists, *‘whose preferred modality for treatment may be a general anaesthetic’* [20]. A more recent shift by Australian paediatric dentists towards the use of DGAs may have occurred. As raised earlier, Alcaino and colleagues noted in 2013 that *‘although most children will cope with dentistry in the normal setting, many may benefit from delivery of extensive dentistry in one session under GA’*. [13]. Cameron, who wrote the corresponding section on GA in the 2008 edition of this reference book, suggested that the need for a DGA is the clinician’s last solution to treating a child’s dental problem [32]. Cameron noted that *‘In most instances, a caring attitude in association with a period of familiarisation will allow the child to be treated conservatively’* and that *‘If, after seeing the child several times, the clinician feels the child needs dental work, but is unmanageable, a general anaesthetic should be considered’*. These comments are more in keeping with UK guidelines [16, 17] but are not included in the 2013 edition of the reference book [13].

Dental hospitalisation is to some extent what investigators of medical practice variation call ‘preference-sensitive’ [33]. Variation may reflect differences in patient or clinician preferences or cost. A 2013 review by the Australian Commission on Safety and Quality in Health Care (ACSQHC) into medical practice variation noted that information asymmetry in health care can be an issue, and that patient preferences can be driven by clinicians. This phenomenon is referred to as ‘supplier-induced demand’ [33]. The other driver of medical care variation identified in the ACSQHC report was ‘supply-sensitive care’ – when more resources, equipment and workforce are available, the more they will be used [33].

These workforce supply factors and information asymmetry between OHPs and parents may be relevant to DGA rates in Victoria. The low oral health literacy levels of some parents were noted by OHP participants as well as the possibility of OHPs using parental guilt that their child has dental caries when discussing the need for a DGA. Also the latest examination of supply and demand in the Australian oral health workforce concluded that there was generally an excess supply of dentists, a shortage of dental therapists, and ‘*no current perceived shortage*’ of paediatric dentists in the private sector in the metropolitan areas with a likely excess of paediatric dentists in the near future except in the public sector and rural and remote areas [34]. The number of registered paediatric dentists in Victoria has increased from four in 1995 to 41 in 2018 [35]. While several participants noted cases where they may have been an element of preference and/or supply sensitive demand in Victoria, the extent of such demand pressures is difficult to quantify and is worthy of further research with larger sample sizes and interviews with paediatric dentists working in the private sector.

Several of the participants mentioned that DGA is convenient for parents because treatment can be completed in one session, rather than three or more visits to a dental clinic. It was noted that DGA can be attractive to parents who are time-poor and asset-rich. Patient/practitioner convenience is considered a contraindication for DGA in the 2017 American APD guidelines for DGA [9]*.* This area is not considered in the Australasian APD 2005 guidelines for children’s DGA. Whether there is a need for new guidelines in Australia will be discussed under policy implications of the study.

All of the OHP participants considered that general dentists may be reluctant to treat young children due to a lack of confidence because they may not have had the necessary training or experience. A general view was that there was not sufficient practical experience during basic dental degree training. Participants also mentioned changes in general dental practice that may have affected DGA rates such as more referral options with increased numbers of registered paediatric dentists and less acceptance by children, parents and dental providers of ‘rough and tumble’ in the dental chair.

### Potential factors to decrease DGA rates

Factors that were identified that are likely to decrease DGA prevalence included: the prevention of dental caries which is the major ‘cause’ of DGAs in children; use of alternatives to DGAs; an appropriate workforce mix; enhancing the oral health literacy of parents; and development and use of evidence-informed guidelines for DGAs. These factors will be reviewed and policy implications considered before discussing the impact of DGAs on health and costs, strengths and limitations of the study, and what further research is required.

All participants agreed that it was preferable to prevent dental caries rather than have to treat it. Primarily this requires addressing the social determinants, that is the ‘upstream’, ‘causes of causes’, such as the political and economic drivers [36]. Addressing sugar consumption is fundamental because sugar is the major proximal cause of dental caries [37]. Other interventions include extending community water fluoridation [38] and using other health professionals such as child health nurses for screening and referral for dental care [39] .

Alternatives to DGAs that participants raised included enhanced child management techniques and use of sedation. A recent pragmatic randomised control trial in Western Australia using dental therapists compared use of atraumatic restorative treatment procedures with the standard care approach for preschool children with dental caries [40]. At 12 months follow up, there was a 45% lower rate of referral for specialist care in the intervention group. These children also reported lower levels of dental fear.

The Dental Board of Australia requires dental practitioners to undertake a training course before they are endorsed to provide intravenous sedation. As noted by a participant, the only Australian course is in Sydney, New South Wales. Just seven Victorian dental practitioners were endorsed in 2018 compared to 49 dental practitioners in New South Wales [41]. There is a need to understand the barriers faced by Victorian practitioners to becoming endorsed. It is likely that training and cost of equipment are key issues.

Several participants commented that a workforce mix is required that provides effective and efficient oral health care to meet community needs. The use of dental and oral health therapists as the primary health care workers with dentists and paediatric specialists available for work outside therapists scope of practice was considered ideal.

Enhancing the oral health literacy of parents would help to address the information asymmetry that exists between them and OHPs and allow for fully informed consent about options to DGA. Poor understanding of the importance of primary teeth and the preventive impact of fluoride were reported by interviewees. A concerning trend was the observed increase in the use of herbal toothpaste rather than fluoride toothpaste.

In relation to DGA guidelines, while all dental clinicians interviewed indicated that a DGA was required for children with high dental treatment needs and/or behavioural issues, views about thresholds varied. Some saw a DGA as the last resort, while others had a lower threshold for hospitalisation. Recently developed clinical guidelines for the Victorian public dental sector support a move away from DGA being the first resort for children for whom dental treatment in a dental chair is not considered possible. A dedicated prevention services appointment is mandated before a DGA service is offered. Recall systems must be implemented for children who do have a DGA [42]. These guidelines also outline what are the most appropriate and long lasting treatment options for a DGA. However the guidelines do not address indications for DGA, and as noted by several participants, there are no standard Australian policy guidelines for referral for publicly funded DGA.

Several paediatric dentists considered that the 2005 Australasian APD guidelines required updating. These guidelines are mostly similar to the 2017 American APD guidelines. A key difference is that there are two additional contraindications for DGA in the latter guidelines: a very young patient with minimal dental needs that can be addressed through therapeutic interventions, and patient/practitioner convenience. New Australian guidelines would need to take into account an enhanced child focus in health care generally, increased DGA safety, treatment shifts, and parent demand.

### Health impacts

Participants considered that the health risks of DGAs were low. Paediatric dentist participants noted that deaths under DGA were rare. Two child deaths under DGA in Australia over the last 20 were discussed. Both deaths were associated with the children’s underlying medical problems. A third child death was raised by one of the participants after the study was completed but no publically available details were found. Child DGA mortality appears to be rare in Australia and lower than the 1:150,000 rate for child GA mortality identified in 2005 [13].

Paediatric dentists indicated that the safety of DGAs had improved with new anaesthetic drugs and better monitoring, a finding supported by an international review of DGAs [43]. Newer anaesthetic agents have fewer side effects and improved monitoring allows problems to be detected earlier when intervention can occur. However nausea was noted by some paediatric dentists as a still common side effect of DGA and could be distressing for the child and parents.

Possible adverse impacts of general anaesthesia on young children’s neuro-development is an active area of research [44]. The United States Food and Drug Administration issued a warning in 2016, updated in 2017, that exposure to anaesthetic and sedative drugs for more than 3 h may cause adverse effects on young children’s developing brain [45]. Children’s DGAs however rarely last longer than half this time [44, 46]. Nelson and Xu in a review of research into the neurological effects of sedation and DGA in children concluded that it is likely to be many years before it is possible to determine the impact on learning and behaviour with confidence [47]. Further research on the side effects of DGAs is required.

### Costs

Based on the conservative assumption that public and private DGAs average $3029 [29], the estimated direct DGA costs for dental Diagnostic Related Groups would have been $571.8 million in Australia in 2013–14. These costs do not include indirect costs such as childcare for other children and loss of income from taking time off work.

### Strengths and limitations

The strength of the qualitative research approach employed in the study was that a detailed exploration of the contributing factors impacting on DGAs was possible. A more comprehensive understanding of decision making contexts related to DGAs was obtained, particularly for factors that are difficult to quantify. Open ended questions allowed participants to describe their perceptions about DGAs. Interviews with HDAMs allowed a deeper understanding of the policy nuances that impact on DGA rates. While the sample was small and targeted, it allowed a set of perspectives on DGAs to be compiled that have not been explored in depth in the literature.

A limitation of the study was that participants worked predominantly in the public sector and although many had also experience in private settings, paediatric dentists working predominantly in the private sector were not interviewed. The key focus for policy and practice implications is therefore in the public sector. Another limitation was that interviews were not audio recorded. While this approach risked loss of data, it was possible to create a more collegial tone between the researcher and participants interviewed, facilitating in-depth discussion about sensitive issues. The principal investigator took notes which were compiled and provided to the participants to check accuracy.

### Research required

The research identified the complex nature of decision making around dental hospitalisation for children at the OHP and parent level. Decisions whether children should receive dental treatment under GA can be complex and raise diagnostic conundrums. The interests of the child, parent and dental provider may align or differ. Issues in the social processes between these participants include power relations, information asymmetry, parental guilt, professional prestige, and commercial pressures. Further research with a medical sociology lens that includes interviewing parents and children would deepen understanding of the complexities related to decision making for DGAs. More research is also required on cost benefit analysis of DGA and alternatives, health and cost impacts, education and workforce issues, and access to operating theatres in the public and private systems. Interviews with private paediatric dentists are required to identify their perspectives on DGAs.

### Policy implications

Policy implications of the study include: expanding courses to train OHPs in alternatives to DGAs; follow up with the family after a DGA to discuss oral hygiene and diet to reduce the likelihood of repeat DGAs for the child or their siblings; address oral health workforce imbalances; enhance families oral health literacy particularly related to the prevention of dental disease and alternatives to DGAs; enhance initiatives to prevent dental caries, the proximal ‘cause’ of most DGAs in children; and develop Australian DGA guidelines.

## Conclusion

The prevalence of hospitalisation of children to treat dental caries is increasing. Many factors influence the prevalence of paediatric dental general anaesthetics – relating to the child, parent, oral health professional, financial impact, health risk, and accessibility to facilities. There are quality of care and convenience benefits but also high costs and possible health risks. Family, workforce and health system factors have been identified that could decrease the prevalence of paediatric dental general anaesthetics.

## Abbreviations

DGAs: Dental general anaesthetics
HADMs: Hospital admission decision makers
OHPs: Oral health professionals
PPDHs: Potentially preventable dental hospitalisations
